# From mechanisms to practice: risk management and multidisciplinary decision-making for systemic treatment-related liver injury in HBV-associated hepatocellular carcinoma

**DOI:** 10.3389/fphar.2026.1871275

**Published:** 2026-09-07

**Authors:** Yachao Tao, Jing Zhou, Yonghong Wang, Yimin Mao, Xuezhong Lei, Enqiang Chen

**Affiliations:** 1 Center of Infectious Diseases, West China Hospital, Sichuan University, Chengdu, Sichuan, China; 2 Department of Cardiology, West China Hospital, Sichuan University, Chengdu, Sichuan, China; 3 Division of Gastroenterology and Hepatology, Renji Hospital, School of Medicine, Shanghai Jiao Tong University, Shanghai Institute of Digestive Disease, Shanghai, China

**Keywords:** hepatitis B virus, hepatocellular carcinoma, immune checkpoint inhibitor, immune-mediated hepatitis, multidisciplinary collaboration, systemic therapy, treatment-related liver injury, tyrosine kinase inhibitor

## Abstract

Hepatocellular carcinoma (HCC) is a leading cause of cancer-related death globally, with chronic hepatitis B virus (HBV) infection as the predominant risk factor. Systemic therapies including tyrosine kinase inhibitors (TKIs), immune checkpoint inhibitors (ICIs), and ICI-antiangiogenic combinations have revolutionized advanced HCC treatment. However, treatment-induced liver injury is a major concern, especially in HBV-related HCC patients with pre-existing inflammation, fibrosis, and cirrhosis, which raises risks of treatment interruption, impaired efficacy, and acute liver failure. Liver injury arises from direct cytotoxicity, immune-mediated hepatitis, vascular damage, and HBV reactivation. This review summarizes the pathophysiological mechanisms of treatment-related liver injury in HBV-related HCC, analyzes the hepatotoxic profiles of tyrosine kinase inhibitors, immune checkpoint inhibitors, combination regimens and emerging agents, and proposes a structured clinical management framework including pretreatment risk assessment, prophylaxis, monitoring, differential diagnosis and severity-based interventions supported by multidisciplinary care. It aims to offer evidence-based guidance for clinicians to preserve liver function while safely administering effective antitumor therapy. The review also highlights key knowledge gaps and future research directions, including predictive biomarkers, personalized risk stratification and proactive safety evaluation of new drugs.

## 1 Introduction

Hepatocellular carcinoma (HCC) remains a major global health burden with high incidence and mortality, and chronic hepatitis B virus (HBV) infection is the leading risk factor, especially in high-prevalence regions including Asia-Pacific and sub-Saharan Africa (Bray et al., 2024; Hwang et al., 2025; Lin and Kao, 2023). HCC treatment has advanced greatly over the past decade: targeted therapy with sorafenib pioneered systemic treatment for advanced HCC, followed by immune checkpoint inhibitors (Huang et al., 2020). Recent immunotherapy-antiangiogenic combinations have further revolutionized care; notably, atezolizumab plus bevacizumab has achieved superior survival benefits and become the standard first-line regimen (Cheng et al., 2022; Finn., et al., 2020a).

Despite improved outcomes, these therapies carry substantial toxicities, with hepatotoxicity as a critical clinical concern—it may necessitate dose adjustments or treatment discontinuation, impair antitumor efficacy, and even trigger acute liver failure (Celsa et al., 2024; Martins et al., 2019; Sangro et al., 2020). This risk is particularly prominent in HBV-related HCC patients, whose livers already suffer from chronic inflammation, fibrosis, cirrhosis and portal hypertension from persistent viral infection. Such pre-existing liver damage compromises hepatic functional reserve and regeneration, heightening vulnerability to anticancer agent-induced injury.

Hepatic injury arises via multiple mechanisms: tyrosine kinase agents (TKIs) directly damage hepatocytes (Paludetto et al., 2019; Zhao et al., 2022), antiangiogenic drugs disrupt intrahepatic hemodynamics and worsen portal hypertension (Ha et al., 2023; Li and Kroetz, 2018), immune checkpoint inhibitors (ICIs) trigger immune-mediated hepatitis by breaking self-tolerance (Hercun et al., 2022; Kumar et al., 2020; Wang et al., 2023). Immunotherapies also carry risks of HBV reactivation—impaired viral immune control drives viral rebound, causing severe hepatitis upon immune restoration (Bengsch et al., 2014; Triantafyllou et al., 2025). Management of HBV-related HCC thus requires balancing effective systemic antitumor therapy with proactive liver protection. This review summarizes the mechanisms, clinical features and management of treatment-induced liver injury to optimize safety and efficacy. It also addresses unresolved diagnostic challenges, discrepancies between trial data and real-world practice, and proposes future research directions to improve the therapeutic index in these complex patients.

## 2 Pathophysiological mechanisms of liver injury in HBV-HCC

Pre-existing liver disease markedly increases mortality from drug-induced liver injury (Chalasani et al., 2015). Treatment-related liver injury in HBV-associated HCC is a complex clinical syndrome, arising from interactions between drug toxicity and pre-existing vulnerable liver microenvironment, and thus the hepatic microenvironment serve as a determinant of injury susceptibility and phenotype (Celsa et al., 2024; Ghabril et al., 2025).

Pre-existing chronic hepatic inflammation, fibrosis and cirrhosis modulate responses to systemic anticancer therapy via interconnected mechanisms. Cirrhosis-related hemodynamic changes and extensive fibrotic tissue alter drug distribution, metabolism and retention, forming localized high-drug-exposure niches that heighten direct hepatotoxicity risks (De Martin et al., 2025; Schrader et al., 2011; Weersink et al., 2020). Persistent viral antigen exposure profoundly reshapes the hepatic immune landscape. In chronic HBV infection, resident liver immune cells such as Kupffer cells and liver-resident memory T cells, often become exhausted or tolerogenic (Bosch et al., 2024; Faure-Dupuy et al., 2019; Han and Shin, 2020; Heim et al., 2024; Provine and Klenerman, 2020). While ICIs reverse T-cell exhaustion to boost antitumor immunity, they disrupt this fragile immune balance—triggering both intended antitumor responses and broken self-tolerance, with non-viral T-cell activation causing bystander hepatitis (Cunningham et al., 2024; Saleh and Elkord, 2019; Shojaie et al., 2021). Besides, fibrotic niches driven by activated hepatic stellate cells recruit regulatory T cells and myeloid-derived suppressor cells, further establishing an immunosuppressive microenvironment (Chou et al., 2011; Kumar et al., 2017).

**FIGURE 1:**
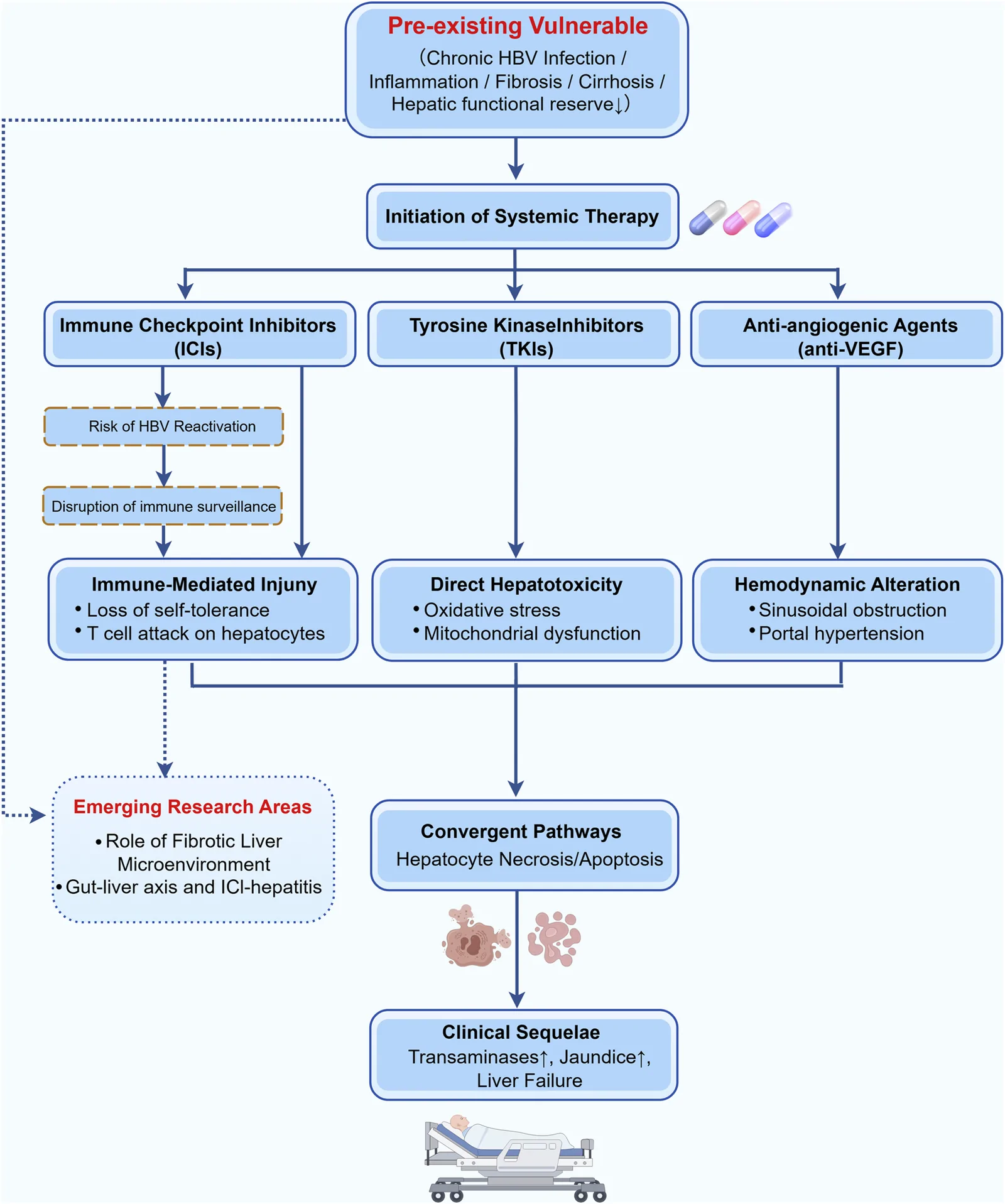
Comprehensive and interlinked mechanisms of liver injury during systemic therapy for HBV-related HCC. This schematic illustrates the multifactorial mechanisms of liver injury during systemic therapy for hepatitis B virus-related hepatocellular carcinoma. The pre-existing liver condition, characterized by chronic inflammation, fibrosis/cirrhosis, and HBV persistence, serves as a vulnerable substrate. Three major therapeutic classes initiate distinct yet potentially synergistic pathological pathways: Tyrosine kinase inhibitors (TKIs) induce direct hepatotoxicity through oxidative stress and mitochondrial dysfunction; immune checkpoint inhibitors (ICIs) trigger immune-mediated hepatitis via T-cell activation and may precipitate HBV reactivation through immune reconstitution; anti-angiogenic agents cause hemodynamic alterations through endothelial injury and exacerbation of portal hypertension. These converging mechanisms ultimately result in hepatocyte necrosis, manifesting clinically as elevated transaminases, jaundice, or liver failure. Emerging research areas, such as the gut-liver axis and the fibrotic liver’s unique immune microenvironment, are highlighted for future investigation.

Drug effects, viral persistence and the hepatic microenvironment jointly drive treatment-related liver injury, which stems from complex host factors rather than direct drug toxicity alone. Clarifying its underlying pathophysiology is critical for accurate diagnosis and targeted management. Figure 1 illustrates how pre-existing liver conditions interact with distinct hepatotoxic mechanisms of TKIs, ICIs and antiangiogenic agents.

### 2.1 Direct hepatocellular toxicity

Direct hepatocellular toxicity is a predictable, dose-dependent intrinsic drug-induced liver injury. Small-molecule agents including TKIs damage hepatocytes by targeting organelles, molecular structures and metabolic pathways via their parent compounds or metabolites. TKI-related liver injury mainly arises from reactive intermediates and disrupted endogenous metabolism. Such intermediates trigger oxidative stress, mitochondrial dysfunction and impaired bile acid homeostasis; they covalently bind proteins and DNA to induce hepatocellular damage and death, while also causing time-dependent inhibition of cytochrome P450 enzymes (LeFort et al., 2024; Paludetto et al., 2019; Rodríguez-Hernández et al., 2020; Saran et al., 2022; Zhao et al., 2022). Histologically, it manifests as focal hepatocellular necrosis with mild inflammation (James et al., 2003). Pre-existing hepatocyte damage, altered drug-metabolizing enzyme activity, and impaired liver regeneration heighten hepatic vulnerability, leading to marked transaminitis even at standard therapeutic doses.

### 2.2 Immune-mediated hepatitis

Immune-mediated liver injury is a critical adverse event of cancer immunotherapy. ICIs disrupt immune homeostasis by blocking programmed cell death protein 1 (PD-1)/cytotoxic T-lymphocyte-associated protein 4 (CTLA-4) to trigger extensive T-cell activation. While boosting antitumor immunity, activated T cells may target hepatocyte self-antigens, causing autoimmune damage to the liver parenchyma (Gudd et al., 2021; Ramos-Casals et al., 2020; Triantafyllou et al., 2025). Histopathologically, lesions vary: interface hepatitis with plasma cell infiltration mimicking autoimmune hepatitis, or lobular hepatitis dominated by abundant CD8^+^ T cells (Cohen et al., 2021; De Martin et al., 2018). In HBV-infected patients, immune interplay grows more complex: ICIs may trigger not only *de novo* immune hepatitis but also reactivate virus-specific T-cell responses, causing severe hepatitis flares with overlapping antitumor, antiviral and autoimmune features (Hutchinson et al., 2021; Llewellyn et al., 2021; Triantafyllou et al., 2025).

Current research on ICI-hepatitis primarily focuses on systemic T-cell overactivation. In chronic HBV infection, however, how treatment-driven immune reconstitution reshapes the hepatic immune microenvironment—including resident memory T cells, Kupffer cells and hepatic stellate cells—and their interactions remains poorly defined (Donne and Lujambio, 2023). Such interplay ultimately determines liver injury susceptibility and phenotypes, and may explain the heterogeneous hepatotoxicity seen in patients receiving identical ICI regimens.

### 2.3 Hemodynamic alterations and vascular injury

Vascular endothelial growth factor (VEGF) has been implicated in pathological angiogenesis associated with tumors, which is mediated by two receptor tyrosine kinases (RTKs), VEGFR-1 and VEGFR-2. Antiangiogenic agents cause distinctive liver injury driven by vascular and hemodynamic changes. VEGF inhibition directly damages hepatic sinusoidal endothelial cells, triggering sinusoidal obstruction, raising intrahepatic resistance and worsening pre-existing portal hypertension (Garbuzenko et al., 2018; Yang et al., 2014; Yao et al., 2023). These drugs also induce systemic hypertension, further disrupting fragile hemodynamic balance in cirrhotic livers and impairing hepatic perfusion (le Noble et al., 2023; Li and Kroetz, 2018). These drugs may also induce a prothrombotic state, raising risks of portal vein thrombosis and acute hepatic decompensation (Fujiwara et al., 2024; Iwakiri and Trebicka, 2021). Such vascular adverse events are particularly hazardous for patients with pre-existing portal hypertension and poor liver functional reserve.

The inhibitory effects of small molecules targeting VEGF or VEGFR on human malignancies have been documented. Bevacizumab, an anti-angiogenic agent targeting VGEF, can disrupt liver microcirculation perfusion and further cause ischemia and hypoxia of hepatocytes by impairing the function of hepatic vascular endothelial cells (Jia et al., 2019; Qu et al., 2024). Almost all TKIs approved for advanced HCC involve the targets of VEGFR (Zheng et al., 2025). For example, Apatinib can competitively bind to VEGFR-2 with high specificity, and It inhibits tumor cell proliferation and blocks tumor angiogenesis, serving as a novel targeted anti-angiogenic agent for HCC (Li H. et al., 2022). Thus, apart from their direct hepatotoxic effects, TKIs may lead to liver injury secondary to vascular and hemodynamic alterations.

### 2.4 HBV reactivation: a critical complication

HBV reactivation is a life-threatening complication in HBV-exposed patients receiving immunosuppressive or immunomodulatory therapy. Sustained viral control relies on intact virus-specific T- and B-cell immune surveillance (Heim et al., 2024; Hoogeveen et al., 2022; Revill et al., 2019; Salimzadeh et al., 2018). Anticancer agents—especially ICIs and corticosteroids for immune-related adverse events—disrupt this balance, enabling viral replication. Subsequent immune restoration triggers severe or fulminant hepatitis flares (Papatheodoridis et al., 2022; Zhong et al., 2022). These findings underscore the critical importance for universal antiviral prophylaxis in this patient population.

### 2.5 The gut-liver axis as a potential modifier

Beyond viral reactivation, the gut-liver axis also modulates treatment-related liver injury. Systemic therapies, especially immunotherapies, disrupt gut microbiota composition and intestinal barrier integrity, increasing translocation of microbial products such as lipopolysaccharides (Gao et al., 2025; Halsey et al., 2020; Lee et al., 2022; McCulloch et al., 2022). These pathogens activate hepatic immune cells including Kupffer cells via portal venous pattern recognition receptors, synergizing with drug-specific effects to aggravate hepatic inflammation (Andrews et al., 2021; Gao et al., 2025). For instance, ICI-induced disruption of gut immune homeostasis may foster a proinflammatory microbiome; its metabolites prime hepatic innate immunity, lowering the threshold for severe ICI-related hepatitis (Llewellyn et al., 2021; Shojaie et al., 2024). Though still preliminary, this mechanism offers a novel angle to explain patient variability and guide liver injury management.

## 3 Hepatotoxicity profiles of contemporary regimens

Liver injury profiles vary greatly across systemic agents and combinations in incidence, onset and manifestations. Recognizing regimen-specific patterns aids toxicity prediction, patient education and personalized monitoring.

### 3.1 Tyrosine kinase inhibitors

As early approved targeted therapies for HCC, TKIs have well-established safety profiles. Sorafenib rarely causes severe hepatotoxicity, mostly presenting as mild, asymptomatic transaminitis; yet rare acute liver failure necessitates close monitoring, especially in patients with impaired baseline liver function, limiting its use to closely observed Child-Pugh A patients (Kelley et al., 2022). Lenvatinib, another first-line TKI, including frequent hypertension—a hemodynamic factor exacerbating liver injury—requiring proactive management (Kudo et al., 2018; Sueta et al., 2017). Later-line TKIs such as regorafenib and cabozantinib share similar safety profiles (Abou-Alfa et al., 2018; Bruix et al., 2017). TKI-related liver injury typically develops within 4–12 weeks of treatment with a clear dose-response correlation, highlighting the need for early, regular monitoring (Huang et al., 2022; Society of Hepatology, 2024).

### 3.2 Immune checkpoint inhibitors

ICIs induce distinct immune-related hepatitis (Cunningham et al., 2024; De Martin et al., 2025). With monotherapy (e.g., ipilimumab, nivolumab, pembrolizumab), the incidence of immune-mediated hepatitis ranges from approximately 5%–10%, and severe (Grade ≥3) events affect 1%–4% of patients (De Martin et al., 2025; Jbag Haanen et al., 2017; LiverTox, 2012). Combination regimens (e.g., ipilimumab plus nivolumab) boost efficacy but markedly increase severe hepatotoxicity—Grade 3–4 events occur in >15% of patients in clinical trials—necessitating prompt, aggressive corticosteroid therapy (Remash et al., 2021; Schneider et al., 2021; Yau et al., 2020). Compared with TKIs, ICI-related hepatitis presents more variable onset, typically 6–12 weeks post-treatment, yet may emerge early after initial dosing or even delayed after discontinuation. Notably, these safety data derive mainly from clinical trials excluding active HBV patients, so real-world risks in HBV-related HCC populations require re-evaluation (Fontana et al., 2023; Liver, 2019; Schneider et al., 2021). For HBV-infected patients, viral reactivation remains critical; though less frequent than with conventional immunosuppressants, its potential severity mandates routine serological screening and universal antiviral prophylaxis before ICI initiation (Loomba and Liang, 2017; Papatheodoridis et al., 2022; Pattullo, 2016).

### 3.3 Combination therapies: ICIs plus antiangiogenic agents

Given the complex pathogenesis of HCC and the immunosuppressive tumor microenvironment, ICIs monotherapy has certain limitations. The response rate to monotherapy with ICIs in HCC is less than 20%, while the efficacy of combination therapy is approximately 30%–36%, rendering combination strategies a more promising therapeutic approach (Tong et al., 2025). Modern first-line HCC therapy commonly combines immunotherapy and antiangiogenic agents. The atezolizumab-bevacizumab regimen exemplifies complex dual hepatotoxicity: immune-mediated injury from anti-PD-L1 blockade and vascular harm from anti-VEGF inhibition, or both (Cheng et al., 2022; Finn et al., 2020a; Lee et al., 2020). Bevacizumab elevates hypertension risk and bleeding from gastroesophageal varices, requiring thorough pretreatment assessment—including upper endoscopy for variceal screening and strict blood pressure control (Ha et al., 2023; Lee et al., 2024; Li and Kroetz, 2018). Regimens like sintilimab plus bevacizumab biosimilar, or durvalumab combined with tremelimumab, share comparable safety profiles (Ren et al., 2021; Sangro et al., 2024). Differentiating immune-mediated from vascular liver injury during elevated liver enzymes remains clinically critical, as it dictates management—immunosuppression or hemodynamic supportive care. Validated noninvasive biomarkers are lacking, compounding this diagnostic challenge. Real-world data show combination regimens carry amplified hepatotoxicity in advanced cirrhosis (Child-Pugh B7–8), raising risks of treatment discontinuation and hepatic decompensation (Pinter et al., 2025). Prospective studies targeting this vulnerable subgroup are urgently needed.

### 3.4 Emerging novel agents

Emerging HCC therapies feature distinct safety profiles requiring rigorous evaluation. TKI–ICI combinations show unique toxicity compared to VEGF/VEGFR antibody–ICI regimens, with frequent total bilirubin elevation (Society of Hepatology, 2024; Zhang et al., 2023). Bispecific antibodies targeting PD-1 and CTLA-4 may cause hepatotoxicity comparable to or exceeding dual ICI regimens (Goebeler et al., 2024; Suzman et al., 2018; Xu et al., 2018). Antibody-drug conjugates (ADCs) cause unique liver injury via cytotoxic payloads, linker instability or antibody targeting, requiring agent-specific assessment (Drago et al., 2021; Wang et al., 2025). Drugs like inotuzumab ozogamicin may trigger sinusoidal obstruction syndrome (SOS), demanding close monitoring (McDonald et al., 2019). For these and other emerging modalities, real-world liver safety data in HBV-related HCC remain limited, highlighting the need for rigorous prospective data collection and post-marketing surveillance.

### 3.5 Limitations of current evidence and real-world disparities

The safety data of systemic therapy mainly derive from clinical trials enrolling Child-Pugh A patients, leaving unclear applicability to real-world Child-Pugh B populations and creating critical evidence gaps (Cabibbo et al., 2024; Pinter et al., 2025). Blindly adopting trial data risks undertreatment from overestimated toxicity and iatrogenic harm. Moreover, ICI-related hepatitis varies markedly among HBV-infected patients, modulated by baseline HBsAg levels, viral genotypes and host genetics—factors rarely assessed in clinical trials. This trial-real world gap creates a key translational challenge, warranting reassessment of current decision-making frameworks—especially for intermediate liver impairment (e.g., Child-Pugh B7) still eligible for systemic therapy (Cabibbo et al., 2024). Such patients require intensified surveillance, closer monitoring, and early intervention even for mild biochemical abnormalities. Future trials should enroll more patients with impaired liver function to generate targeted safety data for this vulnerable group, and well-designed real-world registries are also critical to complement trial evidence and guide routine clinical practice.

To provide a concise and practical summary of the hepatotoxicity profiles discussed in this section, Table 1 offers a comparative overview of the major systemic treatment regimens for HBV-related HCC, delineating their liver injury patterns, overall and Grade 3–4 incidence, and key diagnostic and management considerations. The table highlights marked inter-regimen differences in toxicity risks and phenotypes, emphasizing the need for tailored clinical vigilance.

**TABLE 1:** Comparison of hepatotoxicity risk characteristics of major systemic treatment regimens for HBV-associated HCC.

Treatment regimen	Main type of liver injury	Any grade incidence (approx.)	Grade 3–4 incidence (approx.)	Characteristic manifestation/High-risk factors/Diagnostic notes	Baseline mandatory requirements & considerations
Sorafenib​	Direct Hepatocellular Injury	10∼30% (Kudo et al., 2018, Qin et al., 2021, Qin, Kudo, et al., 2023)	2∼15% (Kudo et al., 2018, Qin et al., 2021)	Dose-related, early onset (within 8-12 weeks).	Child-Pugh A.
Lenvatinib​	Direct Injury + Vascular Effects	10% ∼45% (Ichida et al., 2024 , Kim et al., 2022)	5%∼25% (Kim et al., 2022, Kudo et al., 2018)	Hypertension-related, requires active blood pressure control. Real-world data in Child-Pugh B patients is limited and suggests higher toxicity risk.	Child-Pugh A, proactive blood pressure management. Use with caution in patients with significant portal hypertension.
ICI Monotherapy (e.g., Pembrolizumab)	Immune-Mediated Hepatitis	5∼10% (Finn et al., 2020b, Merle et al., 2023)	1∼4% (Finn et al., 2020b, Zhu et al., 2018)	Median onset 6-12 weeks; can be delayed. irAE pattern. *Note: Trial data from predominantly HBV-negative populations. Real-world incidence in HBV-HCC requires further study. Risk of HBV reactivation necessitates universal prophylaxis.	Child-Pugh A, mandatory prophylactic antiviral therapy. Serial monitoring of HBV DNA is critical.
“T+A” Regimen (Atezolizumab + Bevacizumab)	Mixed Type (Immune + Vascular)	10∼30% (Cheng et al., 2022, Ito et al., 2026, Qin, et al., 2023)	5∼10% (Finn et al., 2020a, Ito et al., 2026)	Primary diagnostic challenge: Urgently differentiate immune-mediated hepatitis (requires corticosteroids) from vascular-mediated injury (requires hemodynamic support). Liver biopsy has high diagnostic value. Real-world studies show higher discontinuation rates due to toxicity in patients with more advanced baseline cirrhosis.	Child-Pugh A, mandatory prophylactic antiviral therapy, upper endoscopy for variceal screening. Aggressive management of hypertension is paramount.
Sintilimab + Bevacizumab Biosimilar​	Mixed Type (Immune + Vascular)	20∼35% (Ren et al., 2021 , Tang et al., 2025)	3∼8% (Ren et al., 2021)	Similar diagnostic dilemma to “T+A” regimen, mandating prompt differentiation between immune and vascular etiologies. Liver biopsy should be considered. Focus on bleeding risk**.**	Child-Pugh A, mandatory prophylactic antiviral therapy, variceal screening.
Dual Immunotherapy (e.g., Durvalumab + Tremelimumab)	Severe Immune-Mediated Hepatitis	20∼40% (Kelley et al., 2021 , Lau et al., 2026)	10∼15% (Kelley et al., 2021)	High incidence and severity of hepatotoxicity, requires readiness for aggressive immunosuppression. Rapid differentiation from other causes (e.g., viral) is essential.	Child-Pugh A, mandatory prophylactic antiviral therapy.
Novel TKI-ICI Combinations (e.g., Lenvatinib + Pembrolizumab)	Predominantly Hepatocellular (Immune component variable)​	30∼50% (Hsu et al., 2024; Llovet et al., 2023; Lv et al., 2024) (Early-phase data)	5∼15% (Llovet et al., 2023) (Early-phase data)​	Early data suggest a high rate of transaminitis, which may be more manageable with dose interruption/reduction than VEGF antibody-based combinations. Long-term safety profile in HBV-HCC is evolving.	Child-Pugh A, mandatory prophylactic antiviral therapy. Requires close monitoring, especially in the first cycle.

* The approximate incidence rates presented in this table are primarily derived from pivotal clinical trials that predominantly enrolled patients with preserved liver function (Child-Pugh class A). In real-world populations, especially those with more advanced underlying cirrhosis (e.g., Child-Pugh class B or C), the incidence and severity of hepatotoxicity may be significantly higher. These data should be interpreted with caution when applied to patients with impaired baseline liver function. HBV: Hepatitis B virus; HCC: hepatocellular carcinoma; ICI: immune checkpoint inhibitor; irAE: Immune-related adverse event; TKI: tyrosine kinase inhibitor; VEGF: vascular endothelial growth factor.

## 4 An integrated framework for clinical management

Safe, effective systemic therapy for HBV-related HCC relies on comprehensive proactive liver safety management throughout treatment, covering pretreatment assessment and timely intervention for confirmed liver injury.

### 4.1 Pre-treatment assessment and risk stratification

A comprehensive baseline assessment forms the cornerstone of management before initiating systemic therapy for HBV-associated HCC. It includes liver functional reserve grading via Child-Pugh, as most systemic agents are indicated only for patients with Child-Pugh class A (Johnson et al., 2022; Lau et al., 2024). Full virological profiling covers HBsAg, anti-HBc, anti-HBs and high-sensitivity HBV DNA testing (Cornberg et al., 2025; Hwang et al., 2020). Contrast-enhanced computed tomography or magnetic resonance imaging evaluates tumor features and chronic liver changes such as nodularity, splenomegaly, portosystemic collaterals and ascites. A detailed clinical history should screen for alcohol use and metabolic steatotic liver disease, while medication review should include prescriptions, over-the-counter drugs and herbal supplements. These elements collectively enable accurate risk stratification prior to treatment initiation.

### 4.2 Universal prophylaxis: the role of antiviral therapy

Post baseline assessment, proactive antiviral prophylaxis is critical for all HBV-exposed patients, including HBsAg-positive, or HBsAg-negative with positive anti-HBc (Sangro et al., 2025). Antiviral prophylaxis should be administered using a nucleos(t)ide analog with a high genetic barrier to resistance, such as entecavir, tenofovir disoproxil fumarate, or tenofovir alafenamide (Cornberg et al., 2025; Sangro et al., 2025; Wong and Lemoine, 2025). Prophylaxis should start 1–2 weeks before systemic therapy and continue for at least 6–12 months afterward, adjusted by serial HBV DNA monitoring (Hwang et al., 2020; Lau et al., 2024; Lau et al., 2021). This strategy effectively prevents the potentially severe complication of HBV reactivation.

### 4.3 Dynamic monitoring during treatment

Upon initiation of systemic therapy, a structured monitoring protocol should be implemented for early detection of liver injury: i) Serum biochemical Monitoring: Perform liver function tests, including alanine aminotransferase (ALT), aspartate aminotransferase (AST), alkaline phosphatase (ALP), gamma-glutamyl transferase (GGT), total bilirubin, albumin, and international normalized ratio (INR), every 2–3 weeks for the first 2–3 months (Brahmer et al., 2018). Extend intervals to 4–6 weeks for stable patients. ii)Virological Surveillance: Monitor HBV DNA every 4–8 weeks to detect breakthrough or reactivation (Ali et al., 2025; Hwang et al., 2020). iii) Imaging Correlation: Use scheduled tumor imaging to assess for hepatic changes, including new/worsening ascites, portal vein thrombosis, or parenchymal abnormalities. iv) Patient Education: Counsel patients to report hepatotoxicity symptoms, such as fatigue, jaundice, dark urine, nausea.

### 4.4 Differential diagnosis of liver injury

Abnormal liver function during systemic therapy requires prompt, systematic evaluation for proper management. Treatment-related liver injury remains a diagnosis of exclusion, necessitating active identification and exclusion of alternative causes. The initial and most crucial step involves exclusion of HBV reactivation through immediate quantitative HBV DNA testing when transaminase elevations are detected. A marked viral load rise confirms reactivation, requiring prompt antiviral intensification and reassessment of immunosuppressive components in the treatment regimen (Lei et al., 2023).

Transaminase fluctuations in patients with HCC are often affected by multiple factors.

Hepatocellular necrosis caused by surgical resection or transarterial chemoembolization (TACE), as well as chemotherapeutic agents administered via hepatic artery infusion chemotherapy (HAIC), such as the FOLFOX regimen, can all lead to elevated transaminase levels. For these patients, hepatoprotective drugs are recommended, as they may normalize transaminase levels.

Other causes require concurrent investigation: repeat cross-sectional imaging rules out tumor progression with biliary obstruction or vascular complications; serologic testing excludes acute viral hepatitis, such as hepatitis A or E virus, cytomegalovirus, or Epstein-Barr virus, especially with acute hepatitis features (Brahmer et al., 2021; Haanen et al., 2022; Kennedy and Salama, 2020). Underlying liver disorders, such as autoimmune hepatitis or metabolic steatotic liver disease, should be evaluated with supportive autoantibody testing as indicated. Full medication review excludes toxicity from concurrent drugs, including antibiotics, antifungals, non-prescription analgesics, or herbal products (Dara and De Martin, 2025; Shatila et al., 2024). In unclear cases, especially severe (Grade≥3) or atypical liver injury, liver biopsy enables definitive pathological diagnosis, distinguishing immune-mediated, viral, sinusoidal, ischemic and granulomatous hepatitis to guide targeted therapy (Brahmer et al., 2021; De Martin et al., 2018; Schneider et al., 2021). Yet its invasiveness limits routine use, highlighting an urgent need for validated noninvasive biomarkers.

**FIGURE 2:**
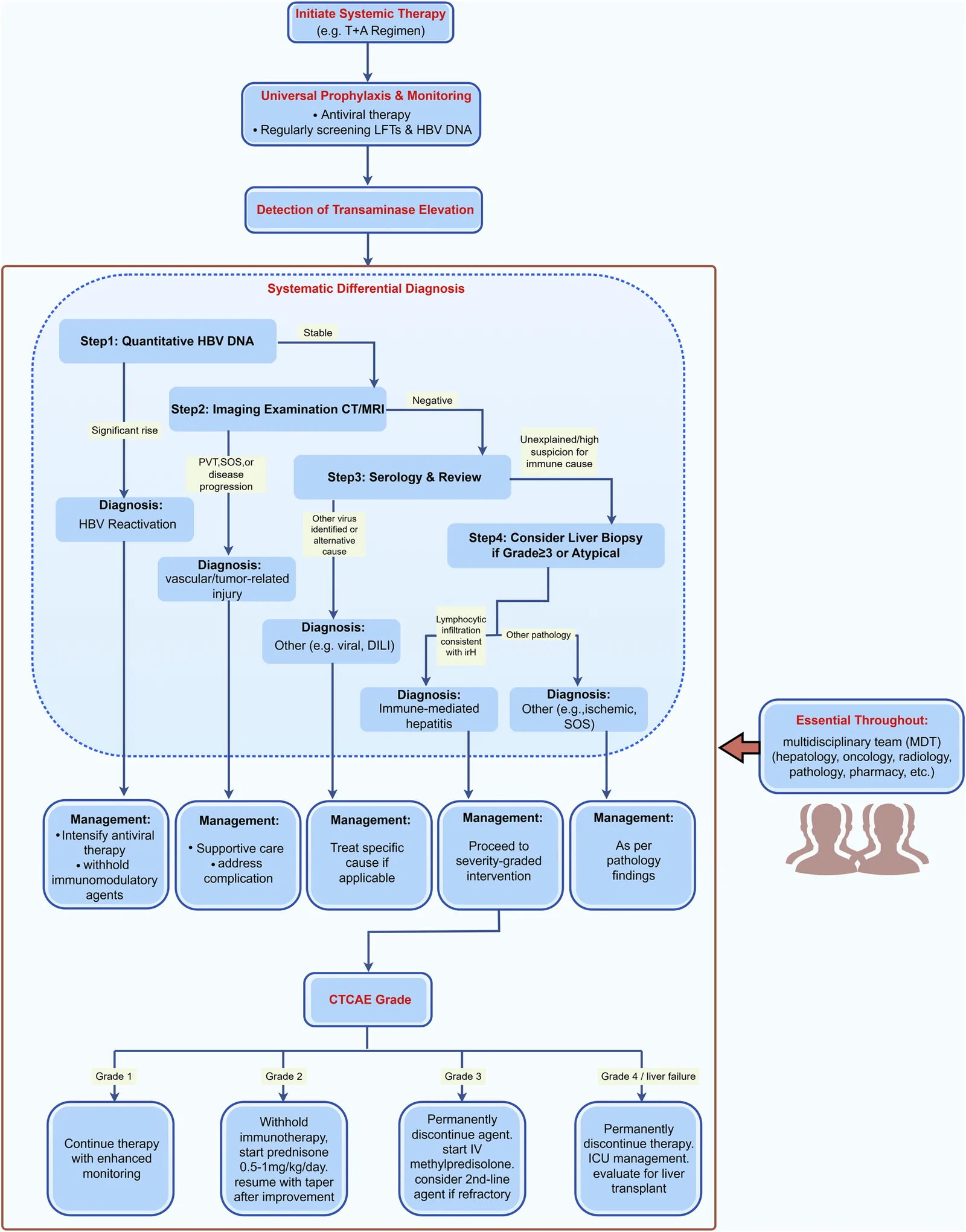
Clinical management algorithm for liver injury in HBV-related HCC patients receiving systemic therapy. This comprehensive algorithm outlines a proactive management pathway, beginning with universal antiviral prophylaxis and structured monitoring. The core of the algorithm is the systematic differential diagnosis workflow (highlighted in blue) for patients developing transaminase elevation, which is critical for combination regimens like atezolizumab + bevacizumab (“T + A″). This sequential process mandates the rapid exclusion of HBV reactivation, vascular complications, and other causes before confirming immune-mediated hepatitis. Liver biopsy is recommended for severe or diagnostically uncertain cases. Management is then tailored to the injury severity (CTCAE grading) and specific etiology, with the imperative that CTCAE grading must be interpreted in the context of the patient’s baseline liver function. A multidisciplinary team (MDT) approach is fundamental to decision-making at every stage.

To address this critical diagnostic challenge and guide clinicians through a systematic evaluation, we propose a structured, sequential diagnostic algorithm (Figure 2). Upon detecting significant transaminases elevation, pomptly exclude HBV reactivation via HBV DNA testing and rule out tumor progression or vascular events with cross-sectional imaging. If initial results remain unclear, differentiate immune-mediated from vascular liver injury using potential biomarkers: cytokine profiles (e.g., elevated CXCL9 and CD163 for ICI-related hepatitis), autoantibody patterns, and contrast-enhanced ultrasound for vascular perfusion changes (Alserawan et al., 2022; Jia et al., 2020; Kang et al., 2021; Nuñez et al., 2023). Integrating clinical, biochemical, serological and imaging data via machine learning also offers a promising direction for future differential diagnosis research.

### 4.5 Severity-graded interventions

Confirmed treatment-related liver injury is managed per CTCAE severity grading (Common Terminology Criteria for Adverse Events v6.0, 2025). This tiered strategy ensures patient safety while sustaining effective anticancer therapy.

Based on CTCAE criteria, graded management for treatment-related liver injury is outlined below (De Martin et al., 2025; Haanen et al., 2022; Peeraphatdit et al., 2020): i) Grade 1 (ALT or AST 1.0–3.0 × the upper limit of normal (ULN); bilirubin 1.0–1.5 × ULN): Continue the drug with enhanced weekly liver function monitoring. ii) Grade 2 (ALT or AST 3.0–5.0 × ULN; bilirubin 1.5–3.0 × ULN): Suspend causative therapy. For immune-mediated hepatitis unimproved after drug withdrawal, give oral prednisone (0.5–1 mg/kg daily). Resume anticancer therapy cautiously upon regression to Grade 1, with steroid tapering over ≥4 weeks iii) Grade 3 (ALT or AST 5.0–20.0 × ULN; bilirubin 3.0–10.0 × ULN): Permanently discontinue the drug; administer high-dose intravenous methylprednisolone (1–2 mg/kg daily). Add second-line immunosuppressants such as mycophenolate mofetil if no improvement within 48–72 h iv) Grade 4 (ALT or AST >20.0 × ULN, bilirubin >10.0 × ULN): Manage emergently in intensive care with high-dose steroids and liver support; conduct urgent multidisciplinary evaluation for liver transplantation.

For HBV reactivation, immediately stop immunomodulators and escalate antiviral therapy. Corticosteroids are controversial and generally avoided, as they may worsen viral replication (Ali et al., 2025; Cornberg et al., 2025). Management should be guided by hepatology specialists experienced in this complication.

### 4.6 Evolving management considerations and unresolved challenges

Though CTCAE guides toxicity grading, its application in HBV-related HCC is complicated by underlying chronic liver disease, as it ignores baseline hepatic reserve and may misjudge clinical severity (Hountondji et al., 2024; Torosian and Dave, 2024). For instance, in Child-Pugh B patients with ascites, an AST >5×ULN without jaundice is rated only Grade 2 per CTCAE. This flaw risks underestimating injury in poor liver function or overrestricting therapy in stable patients with mild baseline abnormalities. Thus, clinical decision-making must integrate CTCAE grades with baseline liver assessments such as Child-Pugh or Albumin-Bilirubin (ALBI) scoring to accurately interpret biochemical changes (Demirtas et al., 2021; Hiraoka et al., 2019; Pinato et al., 2017; Wang et al., 2016).

The implementation of management strategies in HBV-related HCC requires addressing population-specific pathophysiological challenges, especially the safety of corticosteroids use in cirrhosis. While high-dose corticosteroids (e.g., methylprednisolone 1–2 mg/kg/day) treat severe immune-related liver injury, they raise risks of severe infection (such as spontaneous bacterial peritonitis and sepsis), worsened ascites and portal hypertension from fluid retention, and aggravated metabolic disorders including diabetes (Li M. et al., 2022; Pan and Razumilava, 2022). Rigorous benefit-risk assessment is therefore mandatory. For unresolved dosing questions, experts recommend lower initial steroid doses (0.5–1 mg/kg/day) and faster tapering upon improvement in Child-Pugh B patients or those with active comorbidities, alongside strict prophylaxis and multidisciplinary monitoring (Li M. et al., 2022; Zhang et al., 2024).

Beyond steroid optimization, challenges remain in managing steroid-refractory immune-mediated hepatitis in HCC. Evidence for second-line agents like mycophenolate mofetil is limited, while options such as anti-thymocyte globulin and infliximab raise infection risks in cirrhosis (Habib et al., 2025; Ruf et al., 2024; Thompson et al., 2019). Future care requires biomarker-driven precision strategies to predict liver injury risk, differentiate causes, and forecast treatment response. This shift from reactive to personalized proactive management will safeguard the survival benefits of systemic therapy from preventable liver toxicity.

## 5 The central role of multidisciplinary care

**FIGURE 3:**
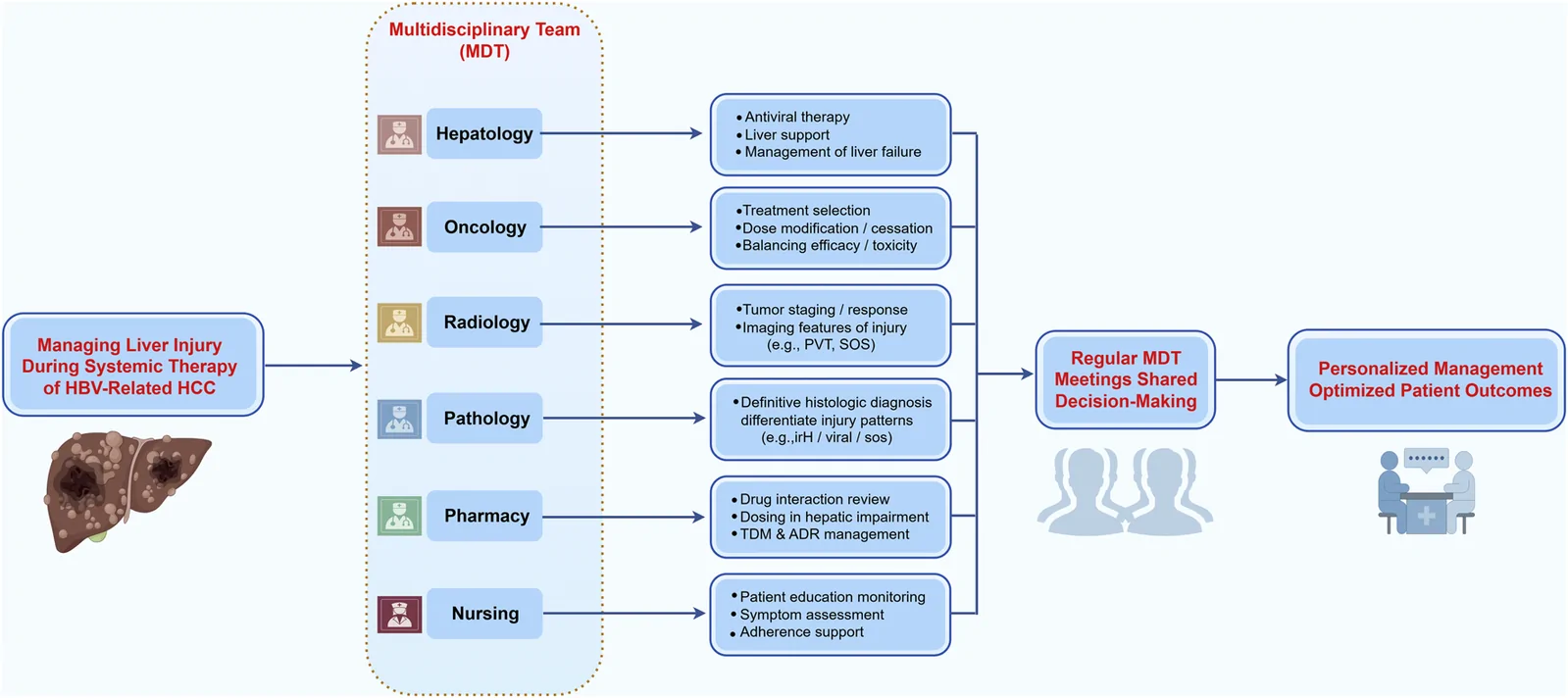
Multidisciplinary collaborative framework for managing liver injury during systemic therapy of HBV-related HCC. This diagram presents the multidisciplinary collaborative framework essential for optimal management of liver injury during systemic therapy for HBV-related hepatocellular carcinoma. The model centers on patient-focused care coordinated through a dedicated multidisciplinary team (MDT) comprising hepatology, oncology, radiology, pathology, pharmacy, and nursing specialists. Each discipline contributes unique expertise: hepatologists manage antiviral therapy and liver support; oncologists optimize treatment strategies and toxicity management; radiologists provide crucial imaging assessment; pathologists offer definitive histological diagnosis; pharmacists ensure drug safety through interaction monitoring; and nursing specialists support patient education and adherence.

Optimal management of systemic treatment-related liver injury in HBV-associated HCC requires a coordinated, multidisciplinary approach (Serper et al., 2017). As shown in Figure 3, this framework centers on close collaboration between hepatologists and oncologists, supported by radiologists, pathologists, clinical pharmacists, and nursing specialists to enable informed, patient-centered decisions (Benson et al., 2021; “European Association for the Study of the Liver. EASL Clinical Practice Guidelines on the management of hepatocellular carcinoma,” 2025). Given the complexity of this clinical issue beyond the scope of any individual specialty, a well-structured multidisciplinary team (MDT) is fundamental for favorable patient outcomes.

### 5.1 Core collaboration: hepatology and oncology

The MDT is centered on close collaboration between hepatologists and oncologists. Oncologists propose antitumor regimens based on disease stage, molecular features and performance status, and adjust doses or modify treatments in response to toxicity. Hepatologists evaluate baseline liver function and portal hypertension, manage antiviral prophylaxis and therapy, diagnose liver injury, and treat severe hepatitis or liver failure, including immunosuppressants for immune-related adverse events (Barone et al., 2013; Marín et al., 2023). This collaborative partnership balances oncologic efficacy and hepatic safety in treatment decisions.

### 5.2 Diagnostic support: radiology and pathology

MDT diagnostic capacity is greatly enhanced by radiologists and pathologists (Nault et al., 2024). Radiologists assist in tumor staging and response evaluation, and detect imaging signs of liver injury such as portal hypertension, vascular thrombosis, and parenchymal changes suggestive of sinusoidal obstruction or ischemic injury. For complex cases, pathologists confirm diagnoses through liver biopsy histology, distinguishing immune-mediated hepatitis, viral hepatitis, SOS, and other drug-induced liver injury to inform treatment.

### 5.3 Medication safety: the clinical Pharmacist’s contribution

Clinical pharmacists are integral to the MDT, improving medication safety by reviewing interactions between anticancer, antiviral, immunosuppressive and supportive drugs. They advise on dose adjustments for hepatic impairment, support therapeutic drug monitoring, and help manage adverse drug reactions. In regular MDT meetings, pharmacists contribute to case discussions and personalized treatment plans, reducing limitations of isolated decision-making.

## 6 Conclusion and future perspectives

Management of treatment-related liver injury in HBV-associated HCC remains a complex and evolving challenge. Modern systemic therapies have improved survival but increased toxicity complexity, and the underlying chronic HBV-related liver disease renders patients highly susceptible to drug-induced liver injury via direct cytotoxicity, immune-mediated mechanisms, vascular damage, and viral reactivation. Addressing this challenge effectively requires a shift from reactive to proactive integrated strategies, including pretreatment risk assessment, universal antiviral prophylaxis, structured monitoring, systematic differential diagnosis, and severity-based algorithms. Successful implementation relies on coordinated multidisciplinary collaboration among hepatologists, oncologists, radiologists, pathologists, and pharmacists.

**FIGURE 4:**
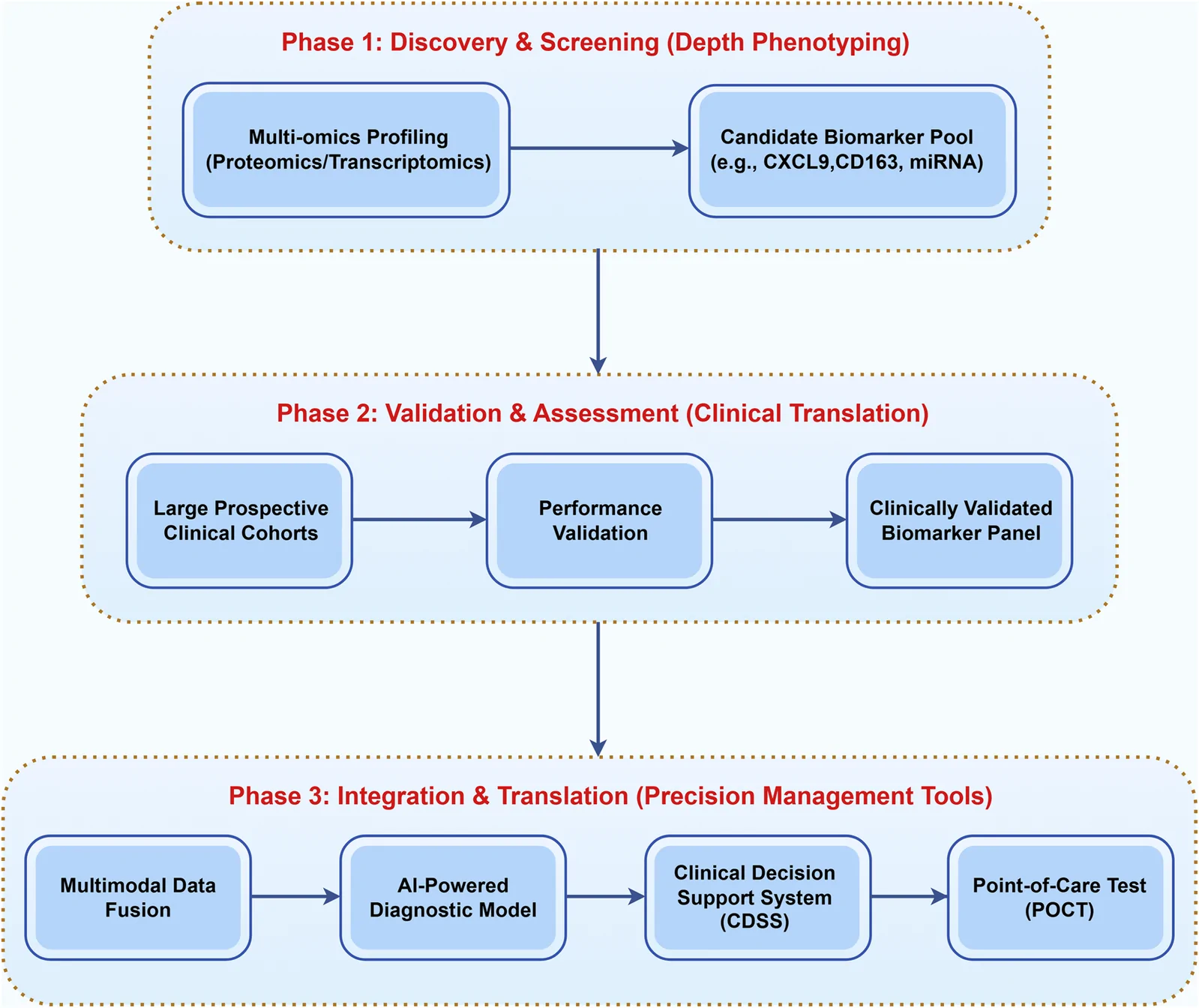
Roadmap for biomarker development and clinical translation in management of systemic treatment-related liver injury in HBV-HCC. This schematic outlines a strategic roadmap to develop and validate novel biomarkers for differentiating the etiology of liver injury (e.g., immune-mediated vs. vascular-mediated) during systemic therapy for HBV-related hepatocellular carcinoma (HCC). The process is divided into three sequential phases: (1) Discovery, utilizing multi-omics profiling of patient biospecimens to identify candidate biomarkers; (2) Validation, assessing the diagnostic performance of these candidates in large, prospective clinical cohorts; and (3) Integration & Translation, combining validated biomarkers with clinical and radiomic data within a machine learning model to create a point-of-care test for rapid, etiology-specific diagnosis and management. The goal is to enable timely and precise interventions, moving beyond the current reliance on clinical exclusion and invasive biopsy. Phase 1: Discovery & Screening (Depth Phenotyping): The initial phase involves unbiased multi-omics profiling (e.g., proteomics, transcriptomics) of biospecimens (serum, tissue) from well-characterized patients who develop distinct types of liver injury during systemic therapy. The objective is to generate a robust Candidate Biomarker Pool (e.g., specific cytokines like CXCL9, CD163, unique autoantibodies, or miRNA signatures). Phase 2: Validation & Assessment (Clinical Translation): Promising candidates from Phase 1 are rigorously validated in large, prospective, multi-center clinical cohorts. Using standardized assays, their diagnostic performance (sensitivity, specificity) and prognostic value for injury severity are confirmed. The output is a clinically validated Biomarker Panel with established cut-off values. Phase 3: Integration & Translation (Precision Management Tools): This final phase focuses on creating practical clinical tools. 1. Multimodal Data Fusion: The validated biomarker panel is integrated with clinical parameters (e.g., Child-Pugh score, treatment regimen) and radiomic features from imaging (e.g., perfusion parameters from contrast-enhanced CT/MRI). 2. AI-Powered Diagnostic Model: Machine learning algorithms are employed to develop an integrated AI Diagnostic Model capable of accurately differentiating injury etiologies and predicting the risk of hepatic decompensation. 3. Point-of-Care Translation: The core biomarkers are developed into rapid Point-of-Care Test (POCT) devices. When combined with the cloud-based AI model, this ecosystem provides a Clinical Decision Support System (CDSS) for real-time, actionable diagnostic and management guidance at the bedside or in the clinic.

Looking ahead, several critical and unresolved issues demand focused attention to refine our management paradigms further (Figure 4). Future directions should prioritize the following areas:i) Developing advanced preclinical models (organoids, humanized mice, computational models) to clarify pathogenic mechanisms. ii) Establishing non-invasive biomarkers and AI-integrated diagnostic algorithms to rapidly distinguish immune-mediated from vascular liver injury, replacing invasive biopsy where possible. iii) Creating personalized risk prediction models combining genetics, viral factors, and tumor features to guide individualized prophylaxis and monitoring. iv) Conducting dedicated trials for understudied populations, especially Child-Pugh B patients and those with steroid-refractory immune-mediated hepatitis or comorbidities. v) Implementing proactive liver safety assessments in trials of novel agents to identify hepatotoxicity early and develop targeted guidelines.

In conclusion, management of treatment-related liver injury in HBV-associated HCC lies at the interface of hepatology and oncology. Translational research, targeted clinical trials in understudied populations, and evidence-based multidisciplinary care can optimize the safety and efficacy of systemic therapies. Shifting from a one-size-fits-all model to personalized strategies will avoid avoidable liver toxicity, preserve survival benefits, and improve long-term outcomes and quality of life for these patients.
